# Editorial: Integrating AI and machine learning in advancing patient care: bridging innovations in mental health and cognitive neuroscience

**DOI:** 10.3389/fmed.2025.1704357

**Published:** 2025-09-29

**Authors:** Salil Bharany, Habib Hamam, SeongKi Kim, Ateeq Ur Rehman

**Affiliations:** ^1^Chitkara University Institute of Engineering and Technology, Chitkara University, Rajpura, Punjab, India; ^2^Faculty of Engineering, University de Moncton, Moncton, NB, Canada; ^3^Department of Computer Engineering, Chosun University, Gwangju, Republic of Korea; ^4^School of Computing, Gachon University, Seongnam-si, Republic of Korea

**Keywords:** artificial intelligence, machine learning, deep learning, mental health, cognitive neuroscience, medical imaging, explainable AI, telemedicine

The overarching goal of this Research Topic is to highlight the transformative potential of artificial intelligence (AI) and machine learning (ML) in enhancing patient care, with a particular focus on mental health and cognitive neuroscience. This Research Topic bridges technological innovations with clinical practice, highlighting state-of-the-art AI and ML models, exploring novel approaches for early detection and monitoring of neurological disorders, emphasizing explainability and trustworthiness in clinical AI, assessing the role of secure infrastructures such as telemedicine and 6G-enabled hospitals, addressing ethical and adversarial concerns, and fostering interdisciplinary collaboration to advance patient-centered healthcare innovation.

The following articles exemplify the diverse applications of AI and ML in healthcare, showcasing innovative approaches that enhance diagnostic accuracy, patient monitoring, and secure clinical practices across various specialties, including mental health, neurology, cardiology, and developmental disorders.

Zhang and Zeng introduced a deep learning-driven image classification model to support mental health diagnostics, addressing the limitations of subjective clinical assessments. By extracting subtle imaging biomarkers from patient data, the model improved diagnostic accuracy and consistency. This approach not only enables earlier detection of psychiatric disorders but also lays the foundation for more personalized treatment strategies. Its impact lies in bridging AI innovations with the urgent needs of mental health care systems.

Shehab and Alhaddad proposed an LSTM-CNN fusion framework for medical image steganalysis, targeting secure telemedicine applications. Their model effectively identified hidden data embedded in medical images, strengthening protection against malicious data tampering. This dual focus on deep learning and cybersecurity ensures trust in digital health platforms. The work is impactful in enabling safe, privacy-preserving telemedicine services as healthcare shifts toward remote and digital care.

Mozhegova et al. evaluated how multimodal AI systems in medicine respond to adversarial perturbations across different input channels. The study revealed key vulnerabilities that could compromise diagnostic integrity, while also offering insights into strategies for resilience. By highlighting the fragility of advanced medical AI under adversarial stress, this work underscores the importance of deploying robust, trustworthy, and secure clinical AI. It sets the stage for developing next-generation defenses against adversarial threats in healthcare.

Ikram et al. harnessed transformer architectures to model sequential ECG signals for arrhythmia detection. Their system outperformed conventional deep learning approaches by effectively capturing long-range dependencies in cardiac patterns. The study demonstrated high diagnostic accuracy, enabling earlier identification of arrhythmias with the potential to prevent severe cardiac events. This represents a major advancement for AI-based preventive cardiology.

Al-Nefaie et al. developed an AI-based diagnostic framework for Autism Spectrum Disorder (ASD), focusing on early and reliable detection. The system integrated multimodal data sources to capture the complex behavioral and neurological patterns associated with ASD. By improving diagnostic speed and reducing reliance on subjective evaluations, the model enhances support for patients and families. This Research Topic highlights the increasing role of AI in addressing neurodevelopmental conditions with significant global health implications.

Together, these articles highlight the practical applications of AI and ML in enhancing patient care. They reveal novel methodologies and intelligent frameworks that improve clinical decision-making, treatment planning, and monitoring across neurological, psychiatric, and other medical domains. By highlighting ethical safeguards, resilience, and secure infrastructures, the collection points to pathways for safe, scalable, and patient-centered healthcare solutions. Overall, the Research Topic illustrates the critical role of interdisciplinary collaboration in translating AI innovations into effective and reliable clinical practice.

